# Oral health in youth with visual impairment: A longitudinal survey

**DOI:** 10.1038/s41598-024-62443-y

**Published:** 2024-05-28

**Authors:** Petra Křížová, Lucie Himmlová, Petr Chramosta, David Vařejčko, Jana Vašáková, Wanda Urbanová

**Affiliations:** 1 Dental Hygiene Study Programme, Third Faculty of Medicine, Charles University, Prague, Czech Republic; 2 Department of Dental Medicine, First Faculty of Medicine Charles University and General University Hospital in Prague, Prague, Czech Republic; 3 Department of Oral, Maxillofacial and Facial Surgery, Regional Health Masaryk Hospital, Ústí Nad Labem, Czech Republic; 4 Department of Stomatology, Third Faculty of Medicine, Charles University and University Hospital Kralovské Vinohrady, Prague, Czech Republic; 5 Faculty of Medicine in Pilsen, Charles University, Pilsen, Czech Republic Prague

**Keywords:** Health care, Medical research

## Abstract

This longitudinal survey aims to demonstrate improvement in oral hygiene among a group of youth with visual impairment (VI) achieved by repeated oral hygiene training, compare their progress with healthy peers (CG) and assess their oral health knowledge. In 100 VI (55♀, 45♂; ± 17.8 years) and 45 CG (23♀, 22♂; ± 17.2 years) oral hygiene training and a Quigley-Hein Plaque Index (QHI) rating were repeated six times at three-month intervals. The VI were divided into four subgroups according to the toothbrush hardness/type. A questionnaire was given to both groups. Appropriate statistical analyses were performed at 5% significance level. Both groups showed reduction in QHI, the VI had overall higher QHI values than CG. Use of an electric toothbrush in VI led to lower QHI in the last examination (p < 0.03). 69% of participants recommended dental specialists to improve communications by acquiring more illustrative aids. VI changed toothbrush less often (p < 0.02). A higher incidence of dental plaque was confirmed in VI compared to CG. After education and individual training, gradual plaque reduction has occurred in both groups. Using an electric toothbrush in VI resulted in better QHI outcomes. Repetitive preventive intervention in youth with VI helped them to adopt healthier oral hygiene habits.

## Introduction

Visually impaired individuals face challenges with everyday skills during childhood, adolescence, and adulthood. Maintaining proper oral hygiene is one of these challenges, especially among adolescents and young adults, when they rely solely on themselves for dental care. The prevalence of visual impairment in children and adolescents in the Czech Republic remains unclear. The available data indicate that the prevalence of childhood blindness in Europe ranges between 0.1 and 0.41 per 1000 children^1^. In a study conducted by Kocur et al.^2^, there were 229 children with severe visual impairments between the ages 6 and 15 years attending all schools for children with visual handicaps in the Czech Republic out of a total of 1,323,578 children of the same age, indicating a prevalence around 0.17 per 1000 children. Little is known about the prevalence of visual impairment in adolescents and young adults in the Czech Republic; according to the Sample Survey of Persons with Health Disabilities from 2019, there were approximately 10,000 individuals between the ages of 15 and 34 with this handicap^3^.

Oral health is more compromised among visually impaired individuals than their sighted peers^4^. They have been shown to have a higher incidence of dental trauma, dental plaque, and calculus accumulation, the occurrence of dental caries, gingivitis, and unfavorable values of oral hygiene indices^4–9^. Adequate dental hygiene can effectively eliminate most of these unfavorable findings in the long term.

Most studies on this topic have been conducted in India, Southwest Asia, Indonesia, and the USA^10,11^. Little is known about the oral health of visually impaired individuals in European countries. Most of the studies have focused solely on assessing the level of oral hygiene and knowledge of oral health. Therefore, our study aimed to demonstrate that long-term improvement in oral hygiene can be achieved among a group of visually impaired youth through individual skill training in oral hygiene and then compare their progress with healthy peers through repeated examinations. Another objective of this study was to assess dental hygiene and oral health knowledge and skills among visually impaired youth and to compare their knowledge, attitudes, and experiences with healthy peers.

## Material and methods

The presented survey was approved by the ethics committee of the Institutional Review Board of Charles University, 3^rd^ Faculty of Medicine. The study was conducted according to the guidelines of the Declaration of Helsinki, and informed consent was provided by the parents/legal representatives or the participants themselves.

A longitudinal intervention survey with a group of youth with visual impairment (VI), running from severe vision impairment to complete blindness, and with healthy peers serving as the control group (CG) was conducted. The VI group consisted of those disabled according to the criteria given by the Persons with Disabilities Equal Opportunities, Protection of Rights and Full Participation Act, 1995; which defines blindness as a condition in which a person suffers from a total loss of vision or visual acuity not exceeding 6/60 or 20/200 in the better eye even with corrective lenses or a visual field limitation of 20° or worse^12^.

An *intervention survey* was conducted on 100 high school youth with visual impairment and 45 healthy youth. In VI there were 55 girls and 45 boys with severe visual impairment or complete blindness, aged 16–21, attending the Gymnázium pro zrakově postižené a střední odborná škola pro zrakově postižené v Praze. The VI was further divided into four subgroups according to the hardness/type of toothbrush they used—15 used a “medium” toothbrush, 28 used a “soft” toothbrush, 30 used an “ultra soft” or “super soft” toothbrush, and 27 used an electric toothbrush. The CG consisted of 23 girls and 22 boys, aged 16–19, from the Gymnázium Ústí nad Labem. The electric toothbrush was used by 16 participants and 34 used manual toothbrushes (23 “soft” and 11 “ultra soft” toothbrushes). Data collection took place between 2016–2020.

All participants were investigated in six school visits at three-month intervals (T_0–_T_6_) by the sole investigator—an experienced dental hygienist—assisted by a trained data recorder. Educational intervention and motivation visits took place with participants seated in their classrooms. Afterward, all participants were individually asked out and seated in a chair in a well-lit place for examinations and further individual oral hygiene training. The plaque on the teeth was visualized by Curaprox PCA 260 Two-Color Plaque Indicator Solution. To conduct a morphometric determination of the amount of dental plaque the plaque scores were recorded using the Quigley and Hein Plaque Index (QHI) as modified by Turesky et al.^13^.

**Figure 1:**
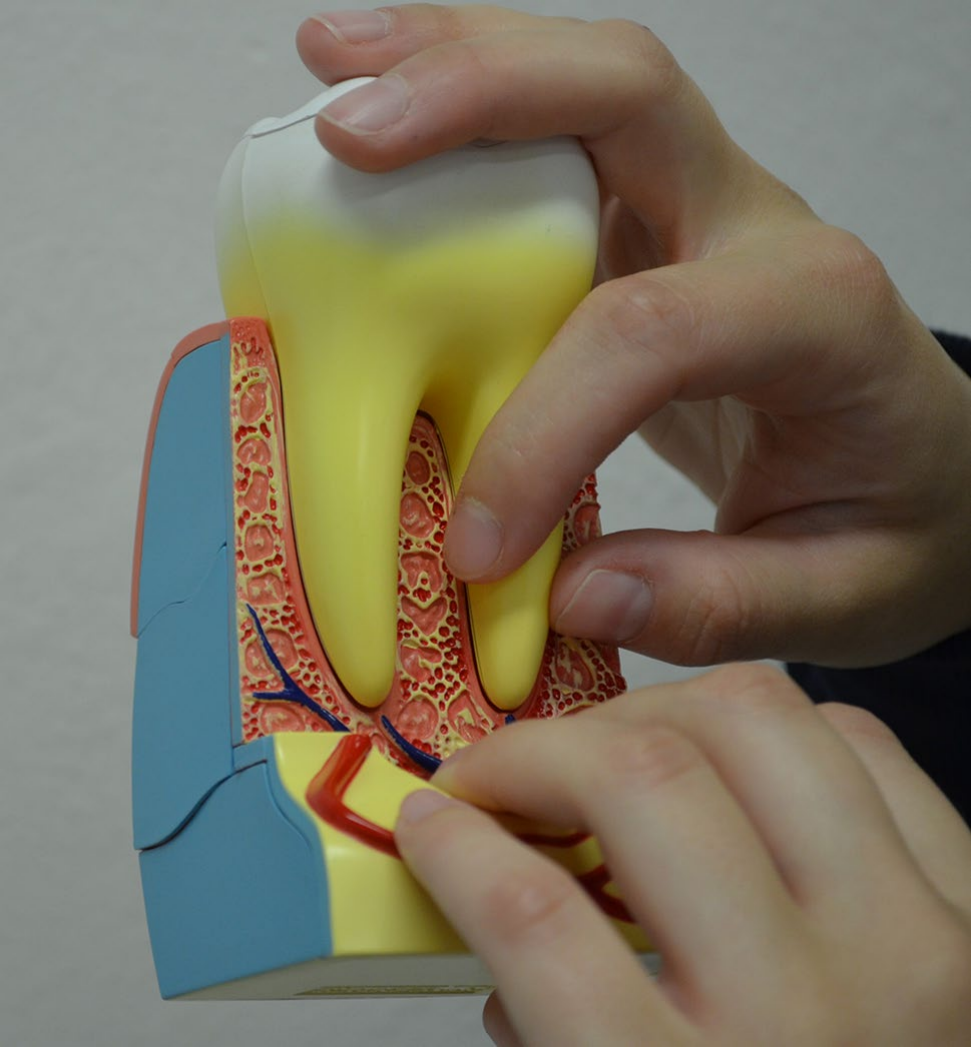
A 3D tooth model allowing the individuals with visual impairment to get an idea of the anatomy of the teeth and alveolar process.

For CG every school visit included group educational intervention and motivation and individual investigation with oral hygiene training. Before the examination, the VI received an additional detailed explanation regarding the instruments and tools used by the examiner. The individual oral hygiene instruction methods used combined audio-tactile performance (ATP) technique, instructions in Braille writing, and 3D tooth models (Fig. 1)^14,15^. The “tell-show-do technique,” recommended for pediatric patients and individuals with disabilities, was used^16^. It involved verbal explanations of procedures in phrases appropriate to the developmental level of the patient, demonstrations of the auditory and tactile aspects of the procedure, and then, without deviating from the explanation and demonstration, completion of the procedure.

The *questionnaire survey* included the same two groups of high school youth. At the beginning of the first school visit, all participants were asked to fill out a questionnaire in written form or the form of a Google questionnaire or the Braille version. Afterward, one investigator entered information from the questionnaires into Excel tables for future processing.

During the first school visit (T_0_), after filling out the questionnaire concerning oral health habits, all participants were asked to brush their teeth with their own toothbrushes as usual. Brushing time was recorded for each individual. Afterward, the QHI values were determined. This was followed by an initial preventive educational intervention, i.e., group education on oral health care, followed by individual practical training in effective oral hygiene. Afterward, a new toothbrush (TePe Select Compact x-soft, soft, medium) or electric brush head (Oral-B Sensitive Clean EB 60–4) and toothpaste with fluoride (elmex® CARIES PROTECTION) were distributed to participants. All participants were instructed on proper toothbrushing duration, technique, and the importance of performing proper dental hygiene every morning and evening. The modified Bass toothbrushing method, consisting of small circles with the toothbrush head at a 45° inclination to the gingival margin, was taught and individually practiced^17^. Toothbrushing times of 3 min with a manual toothbrush and 2 min with an electric toothbrush were applied^18^. It was recommended to check the time intervals by using the stopwatch on a mobile phone or the length of the favorite song. If the participants were already using the interdental cleaning devices, they were encouraged to continue using them; participants who had never used such devices were told to continue their oral health routine as usual. Both groups were instructed on how to identify a properly functioning toothbrush with undamaged fibers. In VI instructions on the quality of the toothbrush must have been done by tactile perception only, they touched a new toothbrush and, for comparison, a toothbrush with damaged and bent fibers unsuitable for further use. For VI instructions were supplemented by teeth models, a model of a tooth with dental caries, a tactile book, a new and used toothbrush for inspection, and text in Braille writing. After a three-month interval, the next school visit (T_1_) was scheduled, including an examination of oral hygiene, QHI individual rating and oral hygiene re-training. At the 6-month school visit (T_2_) QHI values were obtained, and preventive educational interventions with re-education and practical training were performed. Unlike at T0, T1, T3, T4, and T5 participants were not informed about this visit in advance. Interdental aids were added to the home hygiene protocol for all individuals, i.e., interdental silicone toothpicks or flosspicks. All participants were individually instructed in the use of these interdental devices. Further school visits (T_3_–T_5_) were repeated at three-month intervals; all of these were scheduled visits. Repeated investigation of QHI values and preventive intervention with re-education, re-motivation, and practical training were performed on each visit.

IBM SPSS Statistics for Windows, Version 23.0 software (Armonk, NY: IBM Corp) was used for statistical processing of the repeated QHI rating and the questionnaire results at the 5% significance level Appropriate statistical tests were used for each section of the data analysis. Post-hoc power analysis of the study was calculated with Tibico Statistics version 13.4.0.14.

### Ethics approval and consent to participate

The survey was approved by the ethics committee of the Institutional Review Board of Charles University, 3^rd^ Faculty of Medicine. The study was conducted according to the guidelines of the Declaration of Helsinki, a written consent was provided by the parents/legal representatives or the participants themselves.

## Results

The VI and CG groups were compared using Fisher’s exact test for gender and age categories. It was shown that the VI and CG were homogeneous in terms of gender and age. The average age of VI was 17.8 years (SD = 1.4), while for CG it was 17.2 years (SD = 1.0). The CG were significantly younger, with 91% of participants being under 18 years of age, on the contrary, there were only 72% of patients under 18 years of age in VI (p = 0.01).

The average duration of toothbrushing measured at time T_0_ was 74.31 s for the VI (103.2 s for electric toothbrush; 63.6 s for regular toothbrush) and 91.4 s for CG with manual toothbrushes. There was no significant difference between the groups.

### Evaluation of QHI values

**Figure 2:**
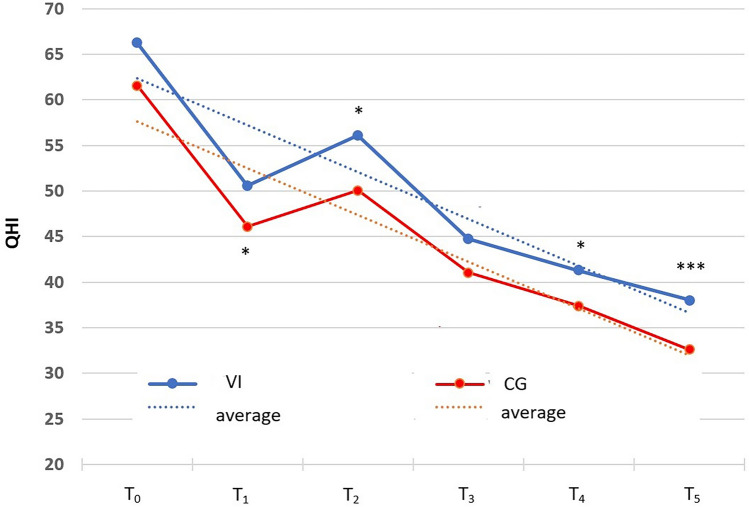
Graphical representation of Quigley-Hein Plaque Index values over time with average trend represented by the linear dotted lines. QHI = Quigley-Hein Plaque Index; T_0-5_ = time of investigation; VI visual impairment group, CG healthy youth; * = p < 0.05; *** = p < 0.001.

The average QHI values for both groups across six measurements are presented in Table 1 and Fig. 2. At T_0,_ the average QHI value for VI was 66.3 and 61.6 for CG. The normality of variable distribution was verified using Shapiro–Wilk tests for all data. If the variable was normally distributed, the groups were compared using the two-sample t-test. If the distribution was not normal, the nonparametric Mann–Whitney U-test was used. The VI had statistically significantly higher plaque index values at T_1_, T_2_, T_4_, and T_5_. There were no statistically significant differences in QHI values between the groups at T_0_ and T_3_.

**Table 1:** Quigley-Hein Plaque Index values (for both groups) for the six examinations performed.

Time of examination	VI					CG					p
	Average QHI	SD	Min	Max	Median	Average QHI	SD	Min	Max	Median	
T_0_	66.3	15.2	26	100	67	61.6	15.1	28	89	65	0.087^a^
T_1_	50.6	13.9	12	78	53	46.1	13.5	20	78	45	0.028*^b^
T_2_	56.1	14.4	18	79	55	50.1	15.0	18	76	53	0.014*^b^
T_3_	44.8	12.3	15	65	45	41.1	11.4	20	65	38	0.075^b^
T_4_	41.3	10.7	15	61	43	37.4	8.9	20	54	39	0.023*^b^
T_5_	38.0	10.2	15	60	37	32.6	7.7	18	49	33	0.0006***^a^

*VI* visual impairment group, *CG* healthy youth.

^a^t-test, ^b^Mann-Whitney U-test.

*p < 0.05.

***p < 0.001.

Comparisons of QHI values between time points (T_0_-T_1_, T_1_-T_2_, T_2_-T_3_, T_3_-T_4_, T_4_-T_5_) were performed using the Wilcoxon test (since the differences did not have a normal distribution). It was demonstrated that both groups experienced statistically significant changes in QHI values for each time interval, showing a decrease in values except for an increase in the T_1_-T_2_ interval (Fig. 2). Both groups of participants showed statistically significant improvement, i.e., a reduction in QHI values between the first and sixth measurements. The two-sample t-test showed that the regression coefficients for participants with visual impairments and the control group did not differ significantly; therefore, improvements in the QHI over time were approximately the same for both groups. However, VI had higher QHI values overall.

Furthermore, in VI, the progression of QHI values was evaluated in relation to the different hardness/types of toothbrushes used. In Table 2, the results of the QHI are presented relative to the four different types of toothbrushes. It was found that VI using an electric toothbrush had lower QHI values at T_5_ (QHI_electric_ = 33) compared to participants using other types of toothbrushes (QHI_manual medium/soft/ultrasoft_ = 38–42; p < 0.03).

**Table 2:** Comparison of Quigley-Hein Plaque Index values in VI relative to the toothbrush type/hardness used.

Time of examination	Toothbrush												p
	Medium			Soft			Ultrasoft			Electric			
	Average QHI	SD	Median	Average QHI	SD	Median	Average QHI	SD	Median	Average QHI	SD	Median	
T_0_	71.8	16.6	69	66.7	15.7	67	68.7	11.1	67	60.1	16.5	62	0.132
T_1_	51.3	12.9	50	49.9	14.7	54	54.2	11.6	55	46.9	15.4	46	0.415
T_2_	56.5	15.5	60	54.4	14.9	55	59.4	12.4	60	54.0	15.4	55	0.548
T_3_	42.7	12.6	44	45.5	12.8	45	47.3	10.9	45	42.3	13.1	44	0.459
T_4_	39.5	9.0	43	41.6	10.4	44	43.6	9.1	44	39.4	13.3	43	0.523
T_5_	38.6	8.3	38	38.1	10.2	37	42.2	9.2	42	33.0	10.5	36	0.029*

*VI* visual impairment group, *SD* standard deviation, *Medium* medium tootbrush hardness, *Soft* soft tootbrush hardness, *Ultrasoft* ultrasoft tootbrush hardness, *Electric* electric toothbrush.

*p < 0.05.

### Questionnaire results

Table 3 summarizes the results from questions regarding visits to the dentist and dental hygienist, performed treatments, and satisfaction with their oral health. Among VI, 34% reported visiting the dentist twice a year, 36% once a year, 15% only when experiencing a problem, and 15% had not visited a dentist. Among CG 15.6% mentioned not visiting the dentist, 45% had no dental issues, and 27.3% did not have a dentist or their dentist was poorly accessible. No significant difference was found between the groups in this regard. Fourteen percent VI reported visiting a dental hygienist 1–2 times a year, 28% had only been to a dental hygiene session once, and 54% had had no dental hygiene appointments. Out of these 54 VI participants four mentioned never hearing about dental hygiene, 26 did not consider it important, and 28 expressed that this treatment was poorly accessible to them. In the control group, 15.6% visited the dental hygienist 1–2 times per year, 24.4% had only been to a dental hygiene session once, and 60% have had no dental hygiene appointments. There was no significant difference in the responses between the groups. In the question “Which dental procedures have you undergone at the dentist”, participants could choose multiple options, and the percentages were calculated based on the total number of answering participants – that is why the sum of percentages does not reach 100%. Most participants (73% VI and 80% CG) selected preventive check-ups as main reason for visiting the dentist, and 96% and 93.3%, respectively, went there for dental filling. Less than half of the participants (48% VI and 37.8% CG) had visited a dentist due to the pain. In the question “Which dental hygienist´s procedures have you undergone”, participants could also choose multiple options. Thirty-two percent VI and 24.4% CG received instruction on dental hygiene with a toothbrush, 31% and 26.7%, respectively, received instruction on interdental aids, and 31% and 20% had undergone teeth polishing. Calculus removal and fluoride varnish application were performed in 8% and 13% VI and 24.4% and 13.3% CG. Most of the participants in both groups expressed that they consider oral health important—87% VI and 75.6% CG. Twenty percent VI and 28.9% CG were fully satisfied with their oral health, while 48% and 33.3%, respectively, acknowledged a need for improvement. Twenty-seven percent VI and 28.8% CG expressed dissatisfaction with the health of their oral cavity.

**Table 3:** Results of the questionnaire regarding visits to the dentist and dental hygienist, treatments received, and level of satisfaction with oral health.

Question	Answer	VI		CG	
		Count	%	Count	%
Do you visit a dentist?	Yes, 2 × a year	34	34.0%	16	35.6%
	Yes, 1 × a year	36	36.0%	18	40.0%
	Only in case of a problem	15	15.0%	4	8.9%
	No, I have not been there for a long time	15	15.0%	7	15.6%
If you do not attend a dentist regularly, please state why	I do not have a dentist	8	26.7%	3	27.3%
	Nothing is bothering me	11	36.7%	5	45.5%
	The dentist is not available	11	36.7%	3	27.3%
Do you visit a dental hygienist?	Yes, 1–2 times a year	14	14.0%	7	15.6%
	Yes, I have already been once	28	28.0%	11	24.4%
	No, I have not been there yet	54	54.0%	27	60.0%
	Other	4	4.0%	0	0.0%
If you do not attend DH, please state the reason	I have never heard of DH	4	6.9%	2	7.4%
	I do not consider it important	26	44.8%	4	14.8%
	DH is hard to find	28	48.3%	21	77.8%
What procedures have you had at the dentist? Why did you visit him/her?	Preventive examination	73	73.0%	36	80.0%
	Dental filling	96	96.0%	42	93.3%
	Pain	48	48.0%	17	37.8%
	Prosthetics	3	3.0%	2	4.4%
	Other reasons	0	0.0%	0	0.0%
What procedures have you undergone at DH? (most common combination a + b + d)	Brushing your teeth with a toothbrush	32	32.0%	11	24.4%
	Demonstration with interdental aids	31	31.0%	12	26.7%
	Removing tartar	8	8.0%	11	24.4%
	Teeth polishing	31	31.0%	9	20.0%
	Fluoridation	13	13.0%	6	13.3%
	I do not know	0	0.0%	0	0.0%
Do you consider oral health important?	Yes	87	87.0%	34	75.6%
	No	8	8.0%	6	13.3%
	I do not know	5	5.0%	5	11.1%
Are you satisfied with your oral health?	Yes	20	20.0%	13	28.9%
	Yes, but it could be better	48	48.0%	15	33.3%
	No, but I would like to improve it	19	19.0%	11	24.4%
	No	8	8.0%	2	4.4%
	I do not know	5	5.0%	4	8.9%

*VI* visual impairment group, *CG* control group, *DH* dental hygienist.

In the Table 4, the responses on communication with the dental specialists are summarized. Thirteen percent VI and 8.9% CG were dissatisfied with how the dental staff communicated with them. No significant difference was found between the responses of the two groups. Both VI and CG recommended improving communication by using more visual/tactile aids (69% VI and 57.8% CG). Among all participants, the most commonly used instructional aides were tooth models and practicing in their mouth (29% and 70% VI and 53.3% and 82.2% CG). Of the 100 VI participants, 17 were educated using a tactile book. When describing the optimal qualities of a dentist, both groups most frequently selected a combination of “communication skills” and “kindness” (45% and 31% VI; 44.4% and 33.3% CG).

**Table 4:** Responses to the questionnaire regarding dentist and dental hygienist communication and the use of instructional aids.

Question	Answer	Group			
		VI		CG	
		Number	%	Number	%
Were you satisfied with the communication of the dentist or dental hygienist?	Yes	29	29.0%	15	33.3%
	Rather yes	58	58.0%	26	57.8%
	Rather no	11	11.0%	4	8.9%
	No	2	2.0%	0	0.0%
How do you recommend improving communication in dental offices?	Improving theoretical knowledge	5	5.0%	2	4.4%
	Improvement of practical skills	16	16.0%	16	35.6%
	Availability of more illustrative aids	69	69.0%	26	57.8%
	Other	13	13.0%	1	2.2%
What tools did they use to demonstrate anatomy and tooth cleaning? (most common a + c combination)	Models of the teeth	29	29.0%	24	53.3%
	Tactile books	17	17.0%	0	0.0%
	Directly in the mouth	70	70.0%	37	82.2%
	Atlas, pictures	15	15.0%	17	37.8%
	None	12	12.0%	11	24.4%
What traits would you like your dentist to have? (most common combination a + c)	Communicative	45	45.0%	20	44.4%
	Less rush	43	43.0%	23	51.1%
	Kindness	31	31.0%	15	33.3%
	Patience	13	13.0%	4	8.9%
	Understanding	9	9.0%	2	4.4%
	Skilled	6	6.0%	1	2.2%
	Cheaper treatments	28	28.0%	15	33.3%
	Individual approach	15	15.0%	7	15.6%
	Stricter	10	10.0%	3	6.7%
	More consistent	0	0.0%	0	0.0%
	Other	0	0.0%	0	0.0%

*VI* visual impairment group, *CG* healthy youth.

Responses to the toothbrushing-related questions (Table 5) were as follows: 89% VI and 88.9% CG reported that their parents taught them how to brush their teeth, while 28% and 33.3%, respectively, learned also from a dentist or dental hygienist, and 11% and 11.1%, respectively, mentioned that nobody taught them how to brush their teeth. Sixty-nine point seven percent VI and 80% CG brushed their teeth twice a day, 19.2% and 15.6%, respectively. Answering the question focused on the duration of toothbrushing the most common response among VI was six minutes (44%), and four minutes (35.6%) among CG. Regarding brushing techniques, most VI preferred circular motions (62%), while CG preferred horizontal motions (80%). Manual toothbrushes and toothpaste were the most commonly used dental hygiene tools (79%/92% VI, 75.6%/91.1% CG); electric toothbrushes were used by 27% VI and 35.6% CG. CG reported changing their toothbrush monthly, while VI participants changed theirs four times yearly. Statistically significant difference between VI and CG was found in the frequency of toothbrush replacement (p < 0.02). Interdental hygiene was performed by 62%VI and 93.3% CG. The frequency of interdental cleaning tool usage among VI participants was “occasional” in 37%, “1–2 times a week” in 19.6% and 28.3%, and “daily” in 15.2%. Among CG, it was “occasional” in 53.3%, “1–2 times a week” in 13.3% and 16.7%, and “daily” in 16.7%. In the question regarding to the toothpaste, 62% VI and 53.3% CG indicated using fluoride toothpaste, while 4% of VI participants and 6.7% CG reported not using toothpaste at all.

**Table 5:** Responses to the questionnaire regarding techniques of toothbrushing and toothbrushing duration.

Question	Answer	Group			
		VI		CG	
		Number	%	Number	%
Who taught you to brush your teeth?	Dentist, hygienist, nurse	28	28.0%	15	33.3%
	Parents	89	89.0%	40	88.9%
	Nobody	11	11.0%	5	11.1%
How many times a day do you brush your teeth?	2x	69	69.7%	36	80.0%
	1x	19	19.2%	7	15.6%
	3 × and more	11	11.1%	2	4.4%
How many minutes do you brush your teeth? (per day)	3	2	2.0%	0	0.0%
	4	14	14.0%	16	35.6%
	5	4	4.0%	8	17.8%
	6	44	44.0%	11	24.4%
	7	10	10.0%	0	0.0%
	8	15	15.0%	4	8.9%
	9	6	6.0%	1	2.2%
	10	5	5.0%	5	11.1%
What technique do you use?	Horizontal	6	6.0%	36	80.0%
	Circular	62	62.0%	7	15.6%
	Sweep technique	19	19.0%	2	4.4%
	Combination	18	18.0%	0	0.0%
	Other	30	30.0%	0	0.0%
How often do you change your toothbrush? *	After a month	13	13.1%	15	33.3%
	Four times a year	82	82.8%	30	66.7%
	Otherwise (how often, why?)	4	4.0%	0	0.0%
What aids do you use? (most common combination a + g)	Toothbrush – manual	79	79.0%	34	75.6%
	Toothbrush – electric	27	27.0%	16	35.6%
	Interdental brushes	22	22.0%	24	53.3%
	Interdental toothpicks, soft-pick	7	7.0%	4	8.9%
	Dental floss	10	10.0%	6	13.3%
	Floss holder, flosspick	23	23.0%	8	17.8%
	Toothpaste	92	92.0%	41	91.1%
	Mouthwash	16	16.0%	5	11.1%
	Other (spray, shower)	9	9.0%	13	28.9%
How often do you use interdental aids?	Occasionally	17	37.0%	16	53.3%
	1–2 times a week	9	19.6%	4	13.3%
	Daily	13	28.3%	5	16.7%
	Every other day	7	15.2%	5	16.7%
What toothpaste do you use?	With fluoride	62	62.0%	24	53.3%
	Without fluoride	17	17.0%	7	15.6%
	I do not know	17	17.0%	11	24.4%
	I do not use any	4	4.0%	3	6.7%

*VI* visual impairment group, *CG* healthy youth.

*p < 0.05.

Additionally, the Spearman correlation coefficient calculation showed that the initial QHI values did not correlate with the measured toothbrushing times for VI (r =  − 0.052) or CG (r =  − 0.140) at T_0_. No correlation of initial QHI values at T_0_ was found in VI or CG participants who regularly use interdental aids compared to those who do not clean interdental spaces. Initial QHI values of participants who visited the dentist regularly twice a year were slightly better in VI participants compared to those who visited less frequently, as evaluated using the Kruskal–Wallis test, however the difference was not significant, and no significant differences were evident in CG. A post-hoc power analysis was performed, calculating the difference between VI and CG based on the QHI values (mean QHI VI = 38; mean QHI CG = 32.6 and shared standard deviation = 9.5 at T_5_). The study had a Type I Error Rate alpha of 0.05. Under these conditions, the test power of the study was determined to be 88.2%.

## Discussion

**Figure 3:**
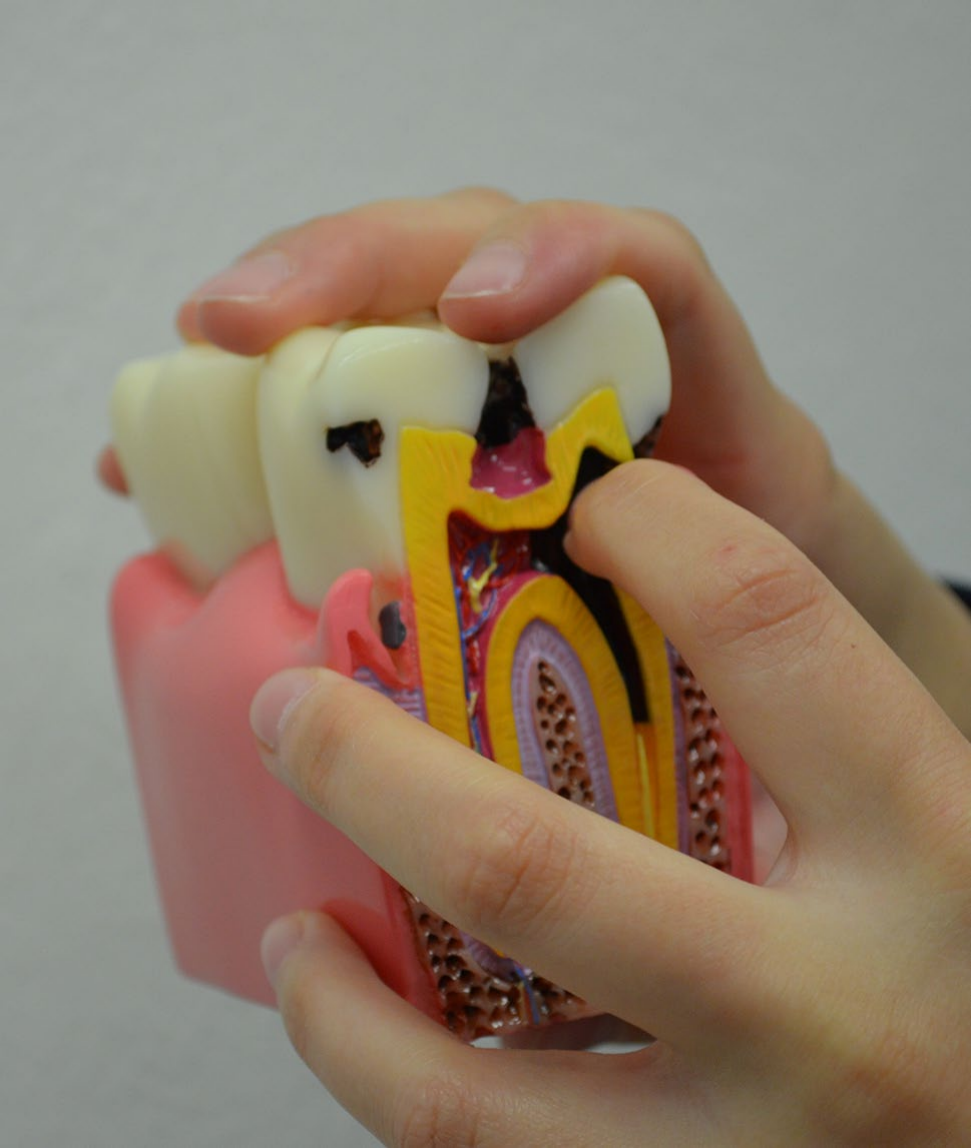
A 3D tactile tooth model used to explain oral diseases such as dental caries and periodontal diseases.

**Figure 4:**
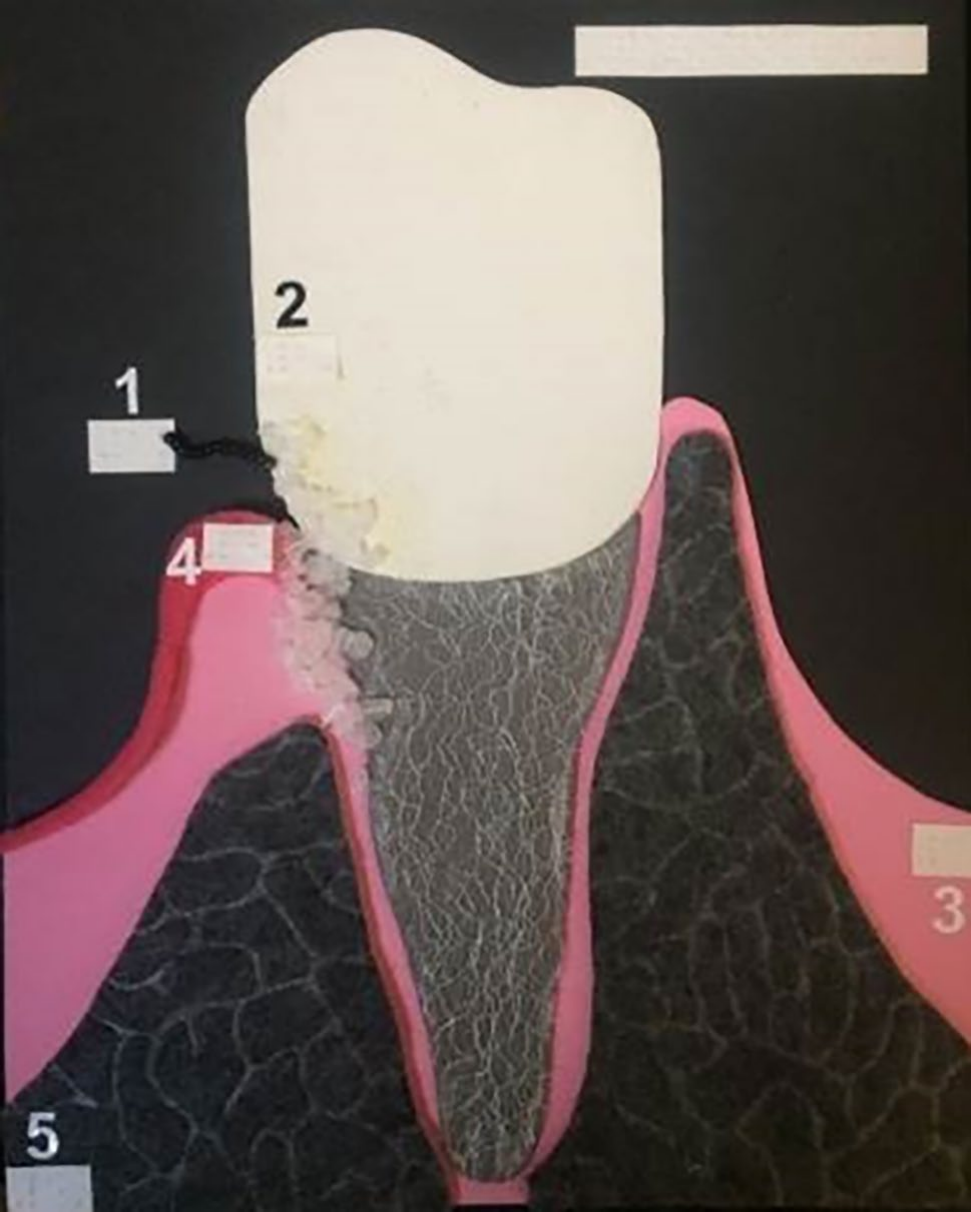
A tactile card created especially for this study to perform symptoms of periodontal disease with instructions in Braille writing depicting following signs: dental calculus, gingival inflammation, gingival recession and bone loss.

**Figure 5:**
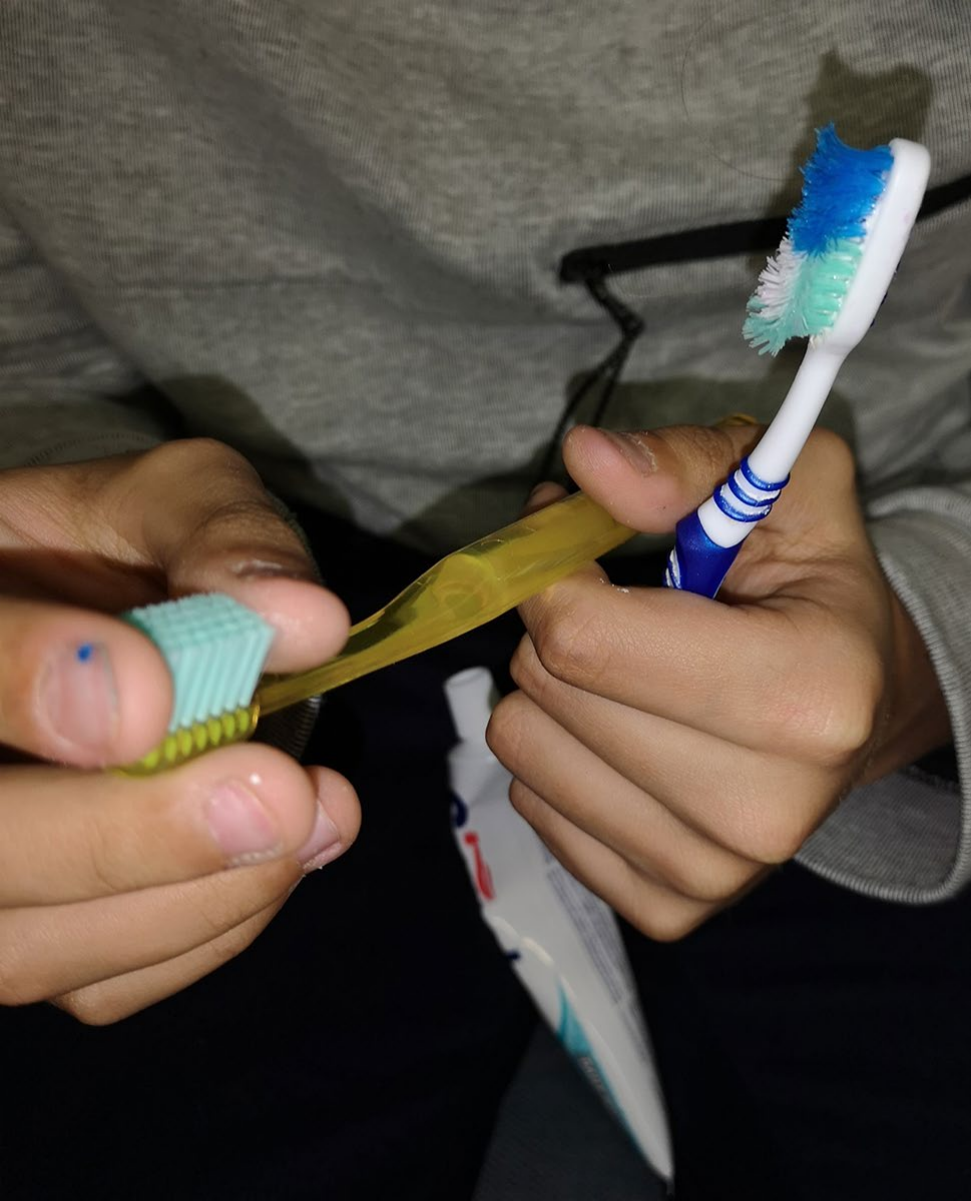
An individual with visual impairment touching a new toothbrush and, for comparison, a toothbrush with damaged and bent fibers unsuitable for further use.

**Figure 6:**
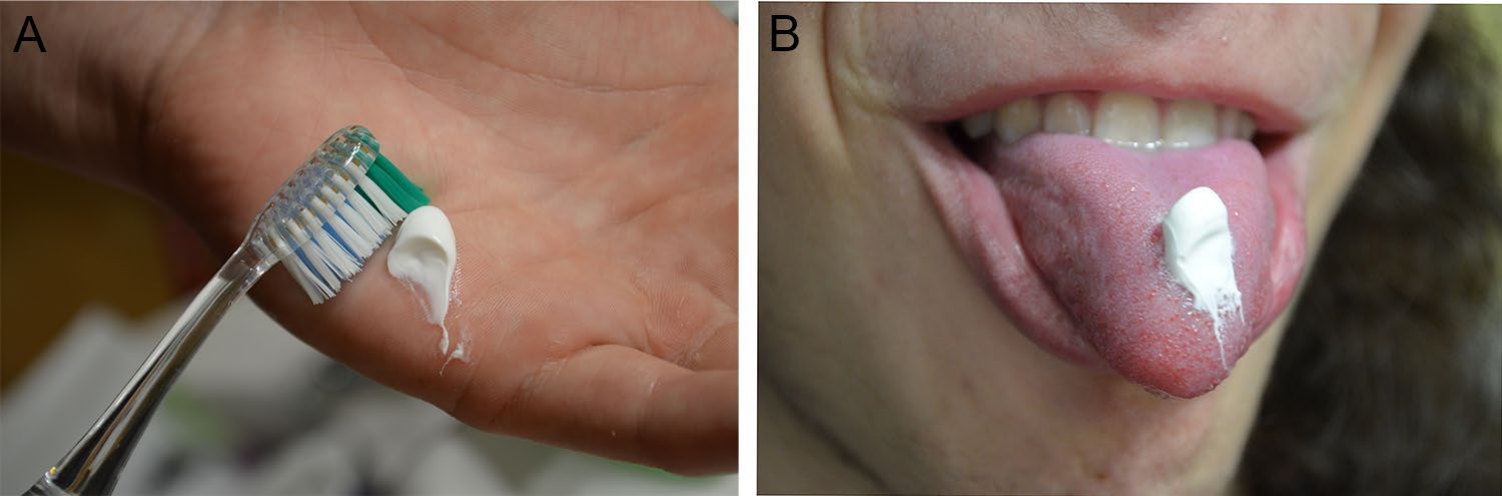
(**A**,**B**) To estimate the right amount of toothpaste in individuals with visual impairment the toothpaste was applied on the palm (**A**) or on the tongue (**B**) and then transferred to the toothbrush.

Visually impaired individuals depend primarily on verbal talk and commands, olfactory sensations, and touch to learn and properly use oral hygiene aids^14^. Working with patients with visual impairments is more challenging for dental hygienists than caring for their sighted peers. The oral hygiene instruction methods reported in the literature that are suitable for subjects with visual handicaps are the use of instructions in Braille writing, audio-tactile performance (ATP) techniques, 3D tooth models, techniques involving tactile or auditory sensations using computer software like Job Access With Speech (JAWSR©) or the use of audio stories^19,20^. In the present study 3D tactile models were used for better explanation of the tooth shape, tooth structure, and diseases such as dental caries and periodontal diseases (Fig. 3). Additional instructional materials were created especially for this study, i.e., tactile cards with dental themes and instructional texts in Braille writing (Fig. 4); as the combination of ATP and Braille proved to be an effective way to improve oral hygiene status in VI^21–25^. Instructions on the quality of the toothbrush and the recommended amount of toothpaste in VI group must have been done by tactile perception. VI had the opportunity to touch and feel a new toothbrush and, for comparison, a toothbrush with damaged and bent fibers unsuitable for further use (Fig. 5). Each participant in the study was given a new toothbrush or an electric toothbrush head at time T_0_ and taught how to compare fiber strength, hardness, and fiber damage so that they could self-assess their toothbrush in the future. The importance of instruction on the quality of brush fibers for VI was evident in the questionnaire, as CG changed their toothbrush significantly more often, visual inspection being probably the primary motivation for changing the toothbrush. The amount of toothpaste was demonstrated to VI by applying the right amount to the palm or tongue and then transferring it to the toothbrush afterward (Fig. 6 A,B), while in CG the proper amount of toothpaste was applied directly to the toothbrush. Effective communication and use of adequate instructional aids regarding dental treatment and oral hygiene instructions was found to be essential in individuals with visual impairment^14^. In the questionnaire almost half of the youth from both groups reported “communication” as one of the main criteria determining their satisfaction with a dental professional. Both VI and CG suggested that dental professionals should obtain additional illustrative aids to improve communication.

The questionnaire showed that more than three-quarters of the subjects from both groups considered their oral health important. However, only one-fifth of them initially brushed their teeth only once a day. Also, measured tooth brushing times averaged about 74 s in VI and 91 s in CG, both being less than the recommended 120/180 s. In the questionnaire, the most frequently reported average tooth brushing time was 3 min twice daily in VI and 2 min twice daily in CG. Since reality differed from the questionnaire results, using timers or a favorite song to set the optimal brushing time was recommended during the first examination.

All participants used a toothbrush, mostly manual, and toothpaste for oral hygiene. Interdental aids were used by 39% of VI and more than three-quarters of CG. This might be one of the reasons for overall better QHI results in CG participants^26^. Positive results of using interdental aids in children with VI were confirmed also in the study of Deepika et al.^27^. However, these small and manipulation-sensitive aids may be challenging and intimidating for VI. Proper motivation and instructions regarding interdental aids led to their effective use in VI at the end of the study.

On the last examination, VI using an electric toothbrush had significantly lower QHI index values than those using manual toothbrushes, regardless of the hardness of the manual toothbrush. This difference may have resulted from the timer on electric toothbrushes and additional movements of the brush head since VI participants using this type of toothbrush had longer actual brushing times and probably more effective brushing technique, even at the beginning of the study. Combined with repeated instruction by the dental hygienist on using an electric toothbrush, the results suggest that an electric toothbrush is more effective in the long term in VI. This finding is in agreement with previous studies where power brushes showed significantly more plaque reduction in VI^28–30^.

The QHI values decreased significantly with each subsequent examination, except for the third examination. The increase between the second and third examinations was probably due to the fact, that participants were not informed about this visit beforehand, while all previous and subsequent examinations were announced in advance. QHI values at T_2_ increased compared to the examination at T_1_ in both VI and CG but did not reach the initial values. At subsequent scheduled visits, a continuous decline in QHI values was noted; however, these positive results may have been partially biased by the participants’ greater efforts to remove plaque deposits prior to the scheduled examination. During our 1.5-year study, the index values decreased by almost half for both groups. The positive effects of repeated instructions on oral hygiene in children and youth with visual impairment were reported in several other studies^21,30–32^. No differences in the decreasing trend of QHI values were found between the VI and CG; but they were lower in the CG at each examination. Repeated instruction and motivation on oral hygiene had an equally positive effect on both VI and CG; however, the healthy participants had overall better oral hygiene. In a study by Yalcinkaya et al.^33^, it was shown that continuous oral hygiene education programs had the same positive effect on children with partial or total visual impairment; therefore, sight seems not to be the main factor in implementing the proper oral hygiene habits.

The questionnaires showed that more than 70% of participants visit the dentist for preventive check-ups at least once a year, however, only a small proportion of them regularly visit a dental hygienist. Initial QHI values of VI who visited the dentist regularly twice a year were slightly better compared to those who visited less frequently, most likely due to taking more care of their oral health in general. Similar dependency was not demonstrated for visits to the dental hygienist, probably because the number of participants regularly visiting was minimal. More than half of the participants in both groups have never been to a dental hygienist. VI reported equally that they did not consider these visits important or had problems finding a dental hygienist, while CG reported that it was more of a problem to find a dental hygienist. Our study demonstrates the benefits of repeated visits to the dental hygienist for the youth—the individual approach and repeated practice in proper tooth brushing technique led to a significant reduction in plaque scores. It would be beneficial to include regular visits to dental hygienists and preventive programs among the basics of preventive care for youth, especially in individuals with special needs^34–36^.

### Limitations

This study has some limitations that should be considered when interpreting the results. Firstly, the subject groups were heterogeneous and nonconsecutive, determined by the school the youth attended, their age, and in the case of VI, also by their disability. Secondly, the assessment of QHI values was not conducted under standardized settings, differences in lighting and noise levels could not be excluded. However, it would have been challenging to conduct a study of this magnitude in a dental office. Finally, it is important to note that positive changes in QHI values may have been partially biased by the participants’ greater efforts to remove plaque deposits prior to the examinations scheduled in advance.

## Conclusion

A higher dental plaque scores were found in participants with visual impairments than in their sighted peers. After collective education, motivation, and repeated individual practice cleaning their teeth, the condition gradually improved in both the VI and CG, however, the QHI values remained better throughout the whole survey in CG. The results suggest that switching to an electric toothbrush has a positive effect on youth with visual impairment. It would be helpful to use more illustrative aids to improve the communication between youth and dental specialists. Repetitive preventive intervention helps young people adopt healthier oral hygiene habits, ultimately promoting better dental outcomes.

## Data Availability

The datasets analyzed during the current study available from the corresponding author on reasonable request.

## References

[1] Gilbert CE, Anderton L, Dandona L, Foster A (2009). Prevalence of visual impairment in children: a review of available data. Ophthal. Epidemiol..

[2] Kocur I, Kuchynka P, Rodný S, Baráková D, Schwartz EC (2001). Causes of severe visual impairment and blindness in children attending schools for the visually handicapped in the Czech Republic. Br. J. Ophthalmol..

[3] Mana M, Chodounská H. Výběrové šetření osob se zdravotním postižením v roce 2018. Web Český statistický úřad. https://www.czso.cz/csu/czso/vyberove-setreni-osob-se-zdravotnim-postizenim-2018. Accesed 15 June 2023. (2019).

[4] Sharififard N, Sargeran K, Katayoun K (2022). Oral health status and related factors in children with visual impairment aged 7–11 years: A cross-sectional study. Front Dent.

[5] Peng B, Petersen PE, Fan MW, Tai BJ (1997). Oral health status and oral health behaviour of 12-year-old urban schoolchildren in the People’s Republic of China. Commun. Dent. Health.

[6] Liu L, Zhang Y, Wu W, He M, Lu Z, Zhang K, Li J, Lei S, Guo S, Zhang Y (2019). Oral health status among visually impaired schoolchildren in Northeast China. BMC Oral. Health.

[7] Dode CB, Cavalcante Y, Risso PA (2022). Traumatic dental injuries and their sequelae in visually impaired adolescents. Dent. Traumatol..

[8] Blanco López MA, Diniz Freitas M, Limeres Posse J, Hernández-Vallejo G, López-Pintor RM (2023). Oral health status and dental care for individuals with visual impairment A narrative review. Spec. Care Dent..

[9] Li J, Zhang K, Cha C, Lu Z, Liu L (2023). Oral health status of students with visual or hearing impairments in Northeast China. BMC Oral Health.

[10] Costa Silva-Freire L, Guimaraes MO, Abreu LG, Vargas-Ferreira F, Vieira-Andrade RG (2022). Oral health issues in children and adolescents with vision impairment: A systematic review and meta-analysis. Int. J. Paediatr. Dent..

[11] Lee JKY, Yuen AWT, Leung KPY, Li JTW, Bae SY, Chan YY, Ip CK, Lau SH, Lau YN, Lo HY, Tang SK, Duangthip D (2024). Oral health status and oral health-related behaviours of Hong Kong students with vision impairment. Healthcare.

[12] Ministry of Social Justice and Empowerment. Government of India. The persons with disabilities (equal opportunities, protection of rights and full participation) Act, 1995. Available from: http://socialjustice.nic.in/pwdact1995.php?pageid=3, (1995).

[13] Turesky S, Gilmore ND, Glickman I (1970). Reduced plaque formation by the chloromethyl analogue of vitamin C. J. Periodontol..

[14] Chowdary PB, Uloopi KS, Vinay C, Rao VV, Rayala C (2016). Impact of verbal, Braille text, and tactile oral hygiene awareness instructions on oral health status of visually impaired children. J. Indian Soc. Pedod. Prev. Dent..

[15] Khan AJ, Ahmad MS, Sabri BAM (2023). The implications of oral health education interventions in providing oral hygiene care for individuals with visual impairment: A systematic review. Spec. Care Dent..

[16] American Academy of Pediatric Dentistry. Behavior guidance for the pediatric dental patient. The Reference Manual of Pediatric Dentistry. Chicago, Ill.: American Academy of Pediatric Dentistry, 321–39. Available from: https://www.aapd.org/research/oral-health-policies--recommendations/behavior-guidance-for-the-pediatric-dental-patient/, (2022).

[17] Smutkeeree A, Rojlakkanawong N, Yimcharoen V (2011). A 6-month comparison of toothbrushing efficacy between the horizontal Scrub and modified Bass methods in visually impaired youth. Int. J. Paediatr. Dent..

[18] Williams K, Ferrante A, Dockter K, Haun J, Biesbrock AR, Bartizek RD (2004). One- and 3-minute plaque removal by a battery-powered versus a manual toothbrush. J Periodontol..

[19] Sardana D, Ritto FP, Ciesla D, Fagan TR (2023). Evaluation of oral health education programs for oral health of individuals with visual impairment: An umbrella review. Spec. Care Dent..

[20] Maulanti T, Nurmala I (2021). A systematic review of oral health educational media innovation for visually impaired children: Which one brings the best impact of change?. Spec. Care Dent..

[21] Tiwari BS, Ankola AV, Jalihal S, Patil P, Sankeshwari RM, Kashyap BR (2019). Effectiveness of different oral health education interventions in visually impaired school children. Spec. Care Dent..

[22] Coutinho DA, Murthy AK, Khond M, Pawar P, Malbhage S, Nikam M (2023). Assessing effectiveness of braille and audio-tactile performance technique in improving oral hygiene status of young adults with visual impairment. Spec. Care Dent..

[23] Bhor KB, Vinay V, Ambildhok K, Shetty V (2021). Effectiveness of oral health educational interventions on oral health of visually impaired school children: A systematic review and meta-analysis. Spec. Care Dent..

[24] Chua H, Sardana D, Turner R, Ting G, Ekambaram M (2021). Effectiveness of oral health education methods on oral hygiene in children and adolescents with visual impairment: A systematic review. Int. J. Paediatr. Dent..

[25] Jawahar A, Maragathavalli G (2021). Audio tactile performance technique as an effective method in improving the oral hygiene status of the visually impaired population in comparison with Braille: A systematic review. J. Res. Med. Dent. Sci..

[26] Londero AB, Reiniger APP, Tavares RCR, Ferreira CM, Wikesjö UME, Kantorski KZ, Moreira CHC (2022). Efficacy of dental floss in the management of gingival health: A randomized controlled clinical trial. Clin. Oral. Investig..

[27] Deepika V, Chandrasekhar R, Uloopi KS, Ratnaditya A, Vinay C, RojaRamya KS (2022). A randomized controlled trial for evaluation of the effectiveness of oral irrigator and interdental floss for plaque control in children with visual impairment. Int. J. Clin. Pediatr. Dent..

[28] Sinha N, Vyas U, Singh B (2021). A prospective case-control study to evaluate oral health status before and after intervention using different dental aids in children with visual impairment. J. Indian Soc. Periodontol..

[29] Sharma A, Arora R, Kenchappa M, Bhayya DP, Singh D (2012). Clinical evaluation of the plaque-removing ability of four different toothbrushes in visually impaired children. Oral. Health Prev. Dent..

[30] Sharififard N, Sargeran K, Gholami M, Zayeri F (2020). A music- and game-based oral health education for visually impaired school children; multilevel analysis of a cluster randomized controlled trial. BMC Oral Health.

[31] Elsman EBM, Al Baaj M, van Rens GHMB, Sijbrandi W, van den Broek EGC, van der Aa HPA, Schakel W, Heymans MW, de Vries R, Vervloed MPJ, Steenbergen B, van Nispen RMA (2019). Interventions to improve functioning, participation, and quality of life in children with visual impairment: A systematic review. Surv. Ophthalmol..

[32] Cui TQ, Lin HC, Lo ECM, Tao Y, Zhou Y, Zhi QH (2017). Randomized clinical trial on the efficacy of electric and manual toothbrushes in plaque removal and gingivitis control in visually impaired school students. Quintess. Int..

[33] Yalcinkaya SE, Atalay T (2006). Improvement of oral health knowledge in a group of visually impaired youth. Oral Health Prev. Dent..

[34] American Academy of Pediatric Dentistry. Adolescent oral health care. The Reference Manual of Pediatric Dentistry. Chicago, Ill.: American Academy of Pediatric Dentistry, 282–91. Available from: https://www.aapd.org/research/oral-health-policies--recommendations/adolescent-oral-health-care/. (2022).

[35] Glassman P, Miller C (2009). Social supports and prevention strategies as adjuncts and alternatives to sedation and anesthesia for people with special needs. Spec. Care Dent..

[36] Vozza I, Cavallè E, Corridore D, Ripari F, Spota A, Brugnoletti O, Guerra F (2016). Preventive strategies in oral health for special needs patients. Ann Stomatol (Roma)..

